# In memoriam: Vladimir Mrša, *professor emeritus*

**Published:** 2024-09

**Authors:** Igor Stuparević, Renata Teparić, Slobodan Barbarić

The progress of research in any scientific field is mostly determined by the work of dedicated and enthusiastic scientists whose high motivation drives the important findings in a particular field. Professor Vladimir Mrša was one such scientist - a highly competent and dedicated researcher who has made a remarkable impact in the field of yeast biochemistry and molecular biology. Over more than four decades, his work has contributed significantly to our understanding of the physiological role of yeast-secreted mannoproteins and the molecular mechanisms of cell wall biogenesis.

Vladimir Mrša passed away on 15 June 2024 at the age of 67 after a courageous battle against cancer. His death came as a shock to many colleagues and friends, as he had actively participated in the Faculty’s anniversary celebrations just a month earlier. As usual, he was in a good mood and engaged in discussion about the state of research at the faculty and other recent news with his colleagues. His death is a great loss for the Faculty of Food Technology and Biotechnology and the University of Zagreb, as well as for the many close colleagues who will remember him as a dear friend.

Vlado was born in Zagreb on 1 June 1957 and completed his primary and secondary education in his hometown. He then decided to study biotechnology at the Biotechnology Department, Faculty of Technology, University of Zagreb, and graduated in 1980 under the mentorship of Professor Blanka Ries. As he demonstrated his talent and high affinity for experimental research while working on his diploma thesis, he decided to pursue postgraduate/PhD study in the field of biochemistry. For his PhD thesis, he worked on the project of Professor Pavao Mildner, Head of the Laboratory for Biochemistry, also under the mentorship of Professor Ries. Mildner's entire research team and other lab members were happy to hear that Vlado would continue working in the lab, since he was not only a talented and competent promising PhD student, but also a very pleasant person. During his work on PhD thesis, Vlado investigated the physiological role of carbohydrate part of yeast mannoproteins, specifically enzymes secreted into the periplasmic space. At that time, the field of glycoprotein research was still in its infancy and it was rather challenging field of research. Vlado was an efficient, hardworking researcher who was fully dedicated to working on his thesis. With the support of his mentor and other senior colleagues who worked on the same project, he successfully carried out the research and defended his PhD thesis at the Faculty of Science, University of Zagreb in 1984.

Prof. Mildner’s laboratory group (from left to right: Branko Kozulić, Vlado Mrša, Ksenija Lopandić, Blanka Ries, Slobodan Barbarić and Pavao Mildner)

Vlado began his academic career in 1985 as a scientific assistant at the Faculty of Food Technology and Biotechnology. Eager to expand his knowledge further, he searched for postdoctoral opportunities abroad and after securing a *Deutscher Akademischer Austauschdienst* (DAAD) (German Academic Exchange Service) fellowship, he joined Professor Widmar Tanner’s laboratory at the University of Regensburg in 1988. Professor Tanner was a leading, world-renowned scientist in the field of yeast glycoproteins. Over the course of two years, Vlado’s research led to impressive results that were published in high-ranking journals. These two years of joint work with Professor Tanner and their mutual appreciation were the basis for a long-lasting collaboration and friendship, and Vlado’s return to Regensburg for further research in 1996 and 1998. The result of their 18-year collaboration were nine joint papers that significantly advanced the understanding of yeast cell wall glycoprotein functions and contributed to the growing knowledge in the field of glycoprotein research. Vlado’s scientific career was greatly enriched by his collaboration with Prof Tanner and earned him an internationally respectable reputation among researchers in the field of yeast molecular biology.

In a later phase of his research career, Vlado focused on studies to develop techniques for the construction of a yeast cell wall display system to immobilise various homologous or recombinant proteins on the surface of the yeast cell wall. Such yeast cell constructs could have great potential for use in various modern biotechnological processes. In the period from 2007 to 2019, he received three consecutive projects funded by the Croatian Science Foundation and carried out in collaboration with his former PhD students Renata and Igor. This joint research was extremely efficient and the obtained results were published in leading journals in the field of applied biotechnology. Today, these colleagues continue this line of research, a testament to the lasting impact of his work.

Prof. Mrša’s laboratory group (from left to right: Vlado Mrša, Antonija Grbavac, Igor Stuparević, Vedranka Fajdetić and Renata Teparić)

As a respected scientist in the field of yeast research, Vlado was an invited lecturer at a number of international meetings and was also instrumental in the organisation of various scientific conferences, including the symposium series *Power of Microbes in Industry and Environment*, which was created as a platform for global scientific dialogue but also as a platform for discussion and exchange of ideas for the development of modern, innovative biotechnologies.

On his journey through the academic ranks — from junior scientist to tenured professor — Vlado had an important influence on the education at the Faculty of Food Technology and Biotechnology. During his academic career, he regularly taught Biochemistry I and II, key courses in all three study programs of the Faculty, as well as specialised biochemistry courses such as Analytical Biochemistry, Protein Purification and Characterisation and Biochemical Function of Vitamins and Ions in Food and Nutrition. He also contributed to the postgraduate and doctoral programs of the Faculty of Food Technology and Biotechnology and the Faculty of Science by teaching several courses and supervising numerous master's and doctoral theses. During work with his postgraduate students, he endeavoured to impart as much knowledge and skills as possible to the students and to inspire the best of them to pursue an academic career. Vlado also had a significant influence on the development of educational programmes at the Faculty of Food Technology and Biotechnology, and even more, he played an important role in shaping the general educational system in Croatia. He coordinated the working group responsible for drafting the chapter on the development of higher education in the *Strategy for Science, Education and Technology of the Republic of Croatia*, which was adopted by the Croatian Parliament in December 2014 and remains the country's most important strategic document for science and higher education.

During his academic career, Vlado held important key positions in faculty management. He was elected Dean of the Faculty of Food Technology and Biotechnology from 2003 to 2007. In addition to his administrative duties, he devoted much of his energy and time to developing the faculty's capabilities to further improve the quality of research work. He was also Vice Dean for International Cooperation. In this role, he intensively promoted international academic partnerships and contributed to programmes such as Erasmus+ and CEEPUS (Central European Exchange Program for University Studies). One of his most important contributions was his dedicated coordination and enthusiastic participation in the implementation of the joint graduate programme between the Faculty of Food Technology and Biotechnology and the Faculty of Science of the University of Zagreb in cooperation with the University of Orléans. He was awarded the prestigious Order of the Academic Palm of the Republic of France for his commitment in promoting this academic cooperation.

As CEEPUS coordinator, Vlado forged strong connections with scientists throughout Central Europe. His honorary membership in the Hungarian Society of Microbiology reflects his close collaboration with Hungarian researchers, especially in the study of yeast cell wall proteins. In addition to his active participation in the *Croatian Microbiological Society*, as a member of Executive Board and the Society's delegate in *Federation of European Microbiology Societies,* he was also a member of various other professional organisations related to the interdisciplinary nature of his field of research. These include the *Croatian Society for Biochemistry and Molecular Biology*, in which he was secretary general and Presidency member, as well as membership in the *Croatian Society for Biotechnology*.

In 2013, Vlado focused on connecting scientific research and industry and joined the Croatian Academy of Engineering (HATZ) as an associate member and was promoted to full member in 2016. He then held key positions such as Secretary-General and was instrumental in increasing the Academy's influence. He was recognised for his leadership, collaboration and tireless commitment to promote the Academy's influence and strengthen the relationship between academia and industry.

Vlado tirelessly advocated for open science and the dissemination of knowledge across borders. He succeeded Professor Mildner as the Editor-in-Chief of *Food Technology and Biotechnology*, an international diamond open access journal published by the Faculty of Food Technology and Biotechnology. Under his leadership since 2009, the journal has thrived and become one of the best scholarly journals in Croatia and beyond, well established in the fields of food science and technology, biotechnology, applied microbiology and industrial engineering.

In 2019, he joined forces with other science editors in Croatia and founded the Croatian Association for Scholarly Communication (CROASC or ZNAK in Croatian). As its first president, he played a vital role in establishing CROASC as the leading scholarly association that promotes open access and provides education and support to science editors and scholarly publishers in Croatia. One of the major activities of the Association is the co-organisation of the PUBMET Conference on Scholarly Communication in the Context of Open Science, which brings together researchers, science editors, librarians, information specialists and others focused on promoting open science and transparent research assessment every year at the University of Zadar.

Vlado in the company of his PUBMET colleagues (from left to right: Dalibor Jakus, Iva Grabarić Andonovski, Lea Škorić, Lovorka Čaja, Vlado Mrša, Ivana Hebrang Grgić, Jelena Viličić, Ivana Končić and Jadranka Stojanovski)

In the course of his scientific and academic career, Vladimir Mrša received numerous awards for his outstanding achievements in research and teaching at the University of Zagreb. In 2006 and 2019, the Faculty of Food Technology and Biotechnology honoured him for ’many years of successful cooperation and outstanding contribution to the promotion of higher education, science and the profession’ and for his ’ultimate scientific contribution and the achieved scientific excellence’. In 1996, the Institute of Cell Biology and Plant Physiology at the University of Regensburg awarded him for the best research results achieved at the institute in that year. The Croatian Society of Biochemistry and Molecular Biology also honoured him for his ’fruitful work within the society and international biochemical associations and for his contributions to the development of Croatian biochemistry and molecular biology." The most notable recognition came after his retirement, when the Senate of the University of Zagreb awarded him the honorary title of *professor emeritus* for his ’extraordinary services to the advancement of the University of Zagreb and his internationally and nationally recognised scientific and educational contribution’. At a small ceremony at the Faculty of Food Technology and Biotechnology in 2023, he proudly thanked the Faculty Council for nominating him and to the University Senate for awarding him this prestigious title.

Despite his many accomplishments, those who knew Vlado best will remember him not only for his sharp intellect and professional achievements, but for his genuine warmth, humility and dedication to the success of others. He was a mentor to many, a colleague who could be relied upon and a friend who brought light to every conversation. His passion for science was matched only by his generosity, and the many students and colleagues whose lives he touched are a testament to his legacy. Above all, Vlado was a devoted husband to his wife Vanja, a loving father to their daughter Lana, and a proud son who cared for his father Zvonimir. His family was the greatest source of his pride and joy.

The passing of *professor emeritus* Vladimir Mrša leaves a deep void in the scientific community, but his contributions—both scientific and personal—will continue to resonate for generations to come. His work has paved the way for future discoveries, and his memory will remain a source of inspiration for all who had the privilege of knowing him. Those of us who worked closely with him for most of his academic career will remember him with great respect and gratitude for his valuable collaboration, thoughtful attitude and pleasant friendship.

Igor Stuparević

Renata Teparić

Slobodan Barbarić

